# Tobacco use and caries increment in young adults: a prospective observational study

**DOI:** 10.1186/s13104-019-4253-9

**Published:** 2019-04-11

**Authors:** Gunnel Hänsel Petersson, Svante Twetman

**Affiliations:** 1 Department of Cariology, Faculty of Odontology, Malmö University, 205 06, Malmö, Sweden; 2 Department of Odontology, Faculty of Health and Medical Sciences, University of Copenhagen, Norre Allé 20, 2200 Copenhagen N, Denmark

**Keywords:** Caries activity, Gender, Smoking, Smokeless tobacco

## Abstract

**Objective:**

Tobacco use has a negative influence on general and oral health but data concerning caries are mainly derived from epidemiological and cross-sectional studies. The aim of this study was to investigate smoking and use of smokeless tobacco (Swedish snus) as determinants of dental caries increment in young adults over 3 years. The baseline cohort consisted of 1295 19-year-olds registered at eight Public Dental Clinics representing socioeconomic strata. After 3 years, 982 of the patients could be reexamined (drop-out rate 24.2%). Caries was scored as decayed and filled surfaces according the WHO criteria and the individual caries increment was recorded by counting the number of surfaces that changed from “sound” to “decayed/filled” over the study period. Information on habitual tobacco use (smoking, snuffing) was collected from a structured questionnaire at baseline.

**Results:**

The baseline prevalence of smoking and use of Swedish snus was 22.3% and 6.3% respectively. Smoking, but not snuffing, displayed a statistically significant relationship with caries increment over 3 years. For smoking, the relative risk was 1.5 (95% CI 1.2–1.7) and the number needed to harm 6.8 (95% CI 4.5–14.2). Thus, habitual smoking is a risk factor for caries in young adults and the findings reinforce arguments that dental health professionals should incorporate anti-smoking activities in their preventive strategies.

**Electronic supplementary material:**

The online version of this article (10.1186/s13104-019-4253-9) contains supplementary material, which is available to authorized users.

## Introduction

It has been suggested that the use of tobacco is associated with impaired oral health and increased risk of dental caries in adolescents and adults [1, 2]. However, in 2013, a systematic review could only identify four published studies on the link between tobacco and caries, all of them assessed of being of low, or very low, quality [3]. Furthermore, data derived from epidemiological and/or cross-sectional studies may establish associations but not causative relationships. Two more recent studies have employed a prospective design; a Swedish study among adolescents found that the caries increment differed significantly between “ever-users” and “never-users” of tobacco but the analyses did not distinguish between smoking and use of smokeless tobacco [4]. The other investigation, conducted in Finnish adults, concluded that daily smoking was independently related to caries development (net DT increment) over a 4-year period [5]. The use of smokeless tobacco (snuff, a mixture of tobacco, water, salt and flavor) needs however further attention since it is a relatively common habit in the Nordic countries, particularly among males. Findings from previous cross-sectional studies have suggested that snuffing was unrelated to caries risk in adults [6, 7] but this has not yet, to the best of our knowledge, been verified in a prospective setting. We have previously validated a computerized caries risk assessment tool in a cohort of young adults [8] and in connection to the baseline examination data on tobacco use was collected but not further analyzed. The aim of the present study was therefore to investigate smoking and smokeless tobacco (Swedish snuff) as determinants of dental caries increment in young adults over a study period of 3 years. The null hypothesis was that neither smoking nor snuffing would be related to caries development.

## Main text

### Materials and methods

The study cohort consisted of all 19-year old patients (n = 1699) registered at eight Public Dental Clinics (PDC) located in the southern Sweden, representing central and rural as well as various socioeconomic strata [8]. Exclusion criteria were planned relocation, leaving the public dental service or severe medical/mental disability. The eligible patients received a written invitation to participate in the project and 76.2% (n = 1295) consented and showed up for the baseline examination. After 3 years, 982 of them could be reexamined (drop-out rate of 24.2%) as presented in a flowchart with reasons for dropping out (see Additional file 1). All participants were residents in communities that were served by piped water with a suboptimal fluoride content (< 0.03 ppm F). The prospective study design was approved by the Ethical Committee; Lund University, Sweden.

#### Clinical examination and data collection

The visual-tactile examinations, including digital bitewing radiographs (two on each side), were performed by the regular oral health team (dentist, dental hygienist, dental assistant) within the Public Dental Service. All teeth were thoroughly dried with compressed air and the scoring was under optimal light with aid of a mirror and explorer. Caries was scored on cavity level according to the WHO-criteria [9] and expressed at the decayed filled surface level (DFS). Caries increment the individual was calculated by counting the number of surfaces that changed from “sound” to “decayed” or “filled” over the study period as well as the progression of an existing caries lesion to a more advanced stage/filling. The radiographs were exposed with aid of a film holder to ensure correct angulation. The responsible dentist evaluated the baseline and follow-up radiographs of each patient, under optimal viewing conditions and proximal lesions involving the dentin were considered as decayed. Hypoplastic or hypomineralized tooth surfaces, mimicking proximal caries, were excluded from the analyses and possible caries reversals were not considered. Prior to the study, each PDC was visited by the principal investigator (GHP) and all participating examiners were trained and calibrated with aid of a series of illustrated clinical cases, including bitewing radiographs, as previously described [8, 10]. Information concerning the habitual use of tobacco at baseline was self-reported through a structured questionnaire. The questionnaire was developed by the health authorities in the Skåne region for mandatorily use at all public dental clinics in order to map the tobacco habits in the region. Occasional smokers (< once a week) were regarded as non-tobacco users and dual users (both smoking and snuffing) were categorized as smokers.

#### Statistical methods

Data were processed with the IBM SPSS software (version 24.0, Chicago, IL, USA). Caries prevalence and incidence was compared with independent samples t-tests. The relative risk (RR) with confidence intervals was calculated from two-by-two tables. P-values less than 0.05 were considered statistically significant. Since the study group was derived from a fixed population-based cohort, no formal power-calculation was carried out prior to the study in order to calculate the sample size.

### Results

The baseline prevalence of habitual tobacco use, smoking and snuffing is shown in Table 1. The baseline proportion of tobacco users was higher among the drop-outs (38.7%) compared with those that remained in the project (25.4%). Smoking was also more prevalent among the females (21.4% vs. 16.1%) while the use of smokeless tobacco dominated among males (12.8% vs. 0.9%). The caries experience at baseline and after 3 years is presented in Table 2. The drop-outs had significantly higher caries rates at baseline compared with the final study group (mean DFS 6.8 vs. 4.3; p < 0.001). The caries burden was significantly higher among the tobacco users compared with subjects without any tobacco habit (p < 0.001). This seemed to be exclusively related to smoking since the caries experience for those using smokeless tobacco (snuff) did not differ from the non-tobacco group. The caries increment over the 3-year observation period is shown in Table 3. Again, habitual tobacco users, and smokers in particular, exhibited significantly more new cavities than the non-tobacco group while the snuffers did not display any increased caries activity. The relative risk for tobacco use was 1.5 with a 95% confidence interval of 1.3–1.8 (p < 0.001). For smokers, the number needed to harm (NNH) was calculated to 6.8 (95% CI 4.5–14.2) which was statistically significant (p < 0.001).

**Table 1:** Baseline prevalence of tobacco use in the study cohort

	Baseline (n = 1295)	Re-examined (n = 982)	Dropouts (n = 313)
Tobacco use	28.6% (24.9/32.6)	25.4% (22.3/28.9)	38.7% (16.0/22.7)
Smoking	22.3% (24.0/20.5)	18.9% (21.4/16.1)	32.9% (15.7/17.3)
Smokeless tobacco (snuff)	6.3% (0.9/12.1)	6.4% (0.9/12.8)	5.8% (0.3/5.4)

Values in table denote total percent with females/males in parenthesis

**Table 2:** Mean caries experience in young adults (n = 1295), expressed as DFS at baseline and after 3 years in relation to tobacco habits (n = 982)

	n	Baseline		3-year follow-up	
		DFS = 0	DFS	DFS = 0	DFS
No tobacco	733	26.7%	4.0 (4.8)	22.9%	5.0 (5.8)
Tobacco use	249	21.3%	5.1^a^ (5.6)	16.9%	6.4^a^ (6.9)
Smoking	186	20.4%	5.6^a^ (5.9)	9.9%	7.1^a^ (7.3)
Smokeless tobacco (snuff)	63	23.8%	3.6 (4.2)	20.6%	4.4 (5.0)

Values in table denote percent, mean and standard deviation (SD)

^a^Statistically significant difference compared with “no tobacco”, p < 0.001

**Table 3:** Caries increment (ΔDFS) in young adults (n = 982) over the 3-year observation period in relation to tobacco habits

	ΔDFS ≥ 0 (%)	ΔDFS	RR	95% CI
No tobacco	32.6	0.9 (1.6)		
Tobacco use	42.2	1.4^a^ (2.4)	1.5^a^	(1.3–1.8)
Smoking	47.3	1.6^a^ (2.6)	1.5^a^	(1.2–1.7)
Smokeless tobacco (snuff)	27.0	0.7 (1.5)	0.8	(0.5–1.3)

The values in the table denote percentage, mean and standard deviation, relative risk (RR) for having new caries lesions and 95% confidence interval (CI)

^a^Statistically significant difference compared with “no tobacco”, p < 0.01

### Discussion

As most previous research on the association between tobacco use and dental caries are based on cross-sectional data, the strength of this present study was its longitudinal design in which the subjects’ tobacco use at baseline could be related to the actual caries development during 3 years. We selected 19-year-olds in order to secure a cohort of homogenous age that could be followed longitudinally for 3 years. This age group of young adults is not yet relocated for studies and/or work and therefore commonly retained within the public dental service. The main findings of this prospective observational study were that smoking but not snuffing showed a significant relationship to caries increment in young adults. The null hypothesis could therefore be rejected for habitual smoking while it was accepted for snuffing. Most important, our prospective results reconfirmed and strengthened previous conclusions obtained from epidemiological and cross-sectional research [3, 7, 11]. Obviously, smoking has a detrimental influence not only on general health but may also increase the burden of caries. However, as caries is a non-communicable disease, it is important to underline that smoking to a large extent may coincide with other biological, behavioral psychosocial and socioeconomic risk factors involved with the disease [12]. For example, smoking can be marker of lifestyle and socio-economy but also exert directly biological influence on salivary buffer capacity and levels of secretory IgA [13, 14].

Smokeless tobacco was in the context of this study exclusive based on Swedish moist snuff (snus) that should not be mixed up with tobacco chewing. The product contains dried tobacco, sodium chloride, water, moisturizers, sodium carbonate and flavor [15]. The finding that the use of snuff was unrelated to caries confirmed those of previous studies [6, 16]. This may be explained by the fact that Swedish snuff products have high pH (7.8–8.5) and may thereby counteract the low pH-conditions in the oral environment that prevail caries lesion formation. Although snuffing seems to be non-cariogenic, it does not rule out the possibility that the habit can be associated with other oral and systemic health hazards [17, 18]. Taken together, our findings reinforce current statements that the members of the oral health team play an important role in individual and population-based antismoking interventions in order to limit the negative influence on oral health [19].

In this study, the prevalence of smoking was higher among young adult females and this was expected in the light of a current report from Sweden [20]. The elevated proportion of smokers that dropped off from the study was however a confounding factor. Since it is most likely that this imbalance have underestimated the true relationship we still are confident with the present findings. Another shortcoming was the relatively large number of examiners and the fact they were not blinded for their patient’s tobacco habits. We tried to overcome this with calibration exercises prior to the investigations and the use of a less sophisticated but well-established caries scoring system. The number of early initial lesions on smooth surfaces and from the radiographs was planned to be scored but as they were found to be inconsistently reported, we refrained to include them in the present report. The data concerning the tobacco use was self-reported and thereby most likely somewhat underestimated. This was however contravened by the fact that prevalence of smoking in the present cohort was much higher than the national average of 10–14% [21]. In further longitudinal trials, our best suggestion would be to (i) score the smoking/tobacco use in more detail (frequency, amount, duration, debut age etc.), and, (ii) add proximal initial caries lesion progression as indicator of caries activity.

### Conclusion

Habitual smoking, but not snuffing, showed a significant relationship to caries increment over a 3-year period in young adults. The findings reinforce arguments that dental health professionals should incorporate anti-smoking activities in their preventive toolbox to improve oral and general health.

## Limitations

In this cohort, smoking was a significant determinant for caries development in young adults but being a complex multifactorial disease, other biological, behavioral psychosocial and socioeconomic risk factors, not assessed in this paper, are also involved in the course of the disease. Although all examiners in this study were thoroughly calibrated with respect to caries scoring, the inter-examiner agreement in terms of Kappa-values was not tested. A certain impact on the registrations cannot therefore be ruled out.

## Additional file

**Additional file 1.** Flow-chart of the 3-year study with reasons for dropping out.

## Abbreviations

WHO: World Health Organisation
PDC: Public Dental Clinic
DFS: decayed filled surfaces
RR: relative risk
CI: confidence interval
