# Supplementary figures and images for: Tobacco use and caries increment in young adults: a prospective observational study
Source: BMC Res Notes. 2019 Apr 11;12:218. doi: 10.1186/s13104-019-4253-9 (PMC6458795; doi:10.1186/s13104-019-4253-9)

**Additional file 1.** Flow-chart of the 3-year study with reasons for dropping out.

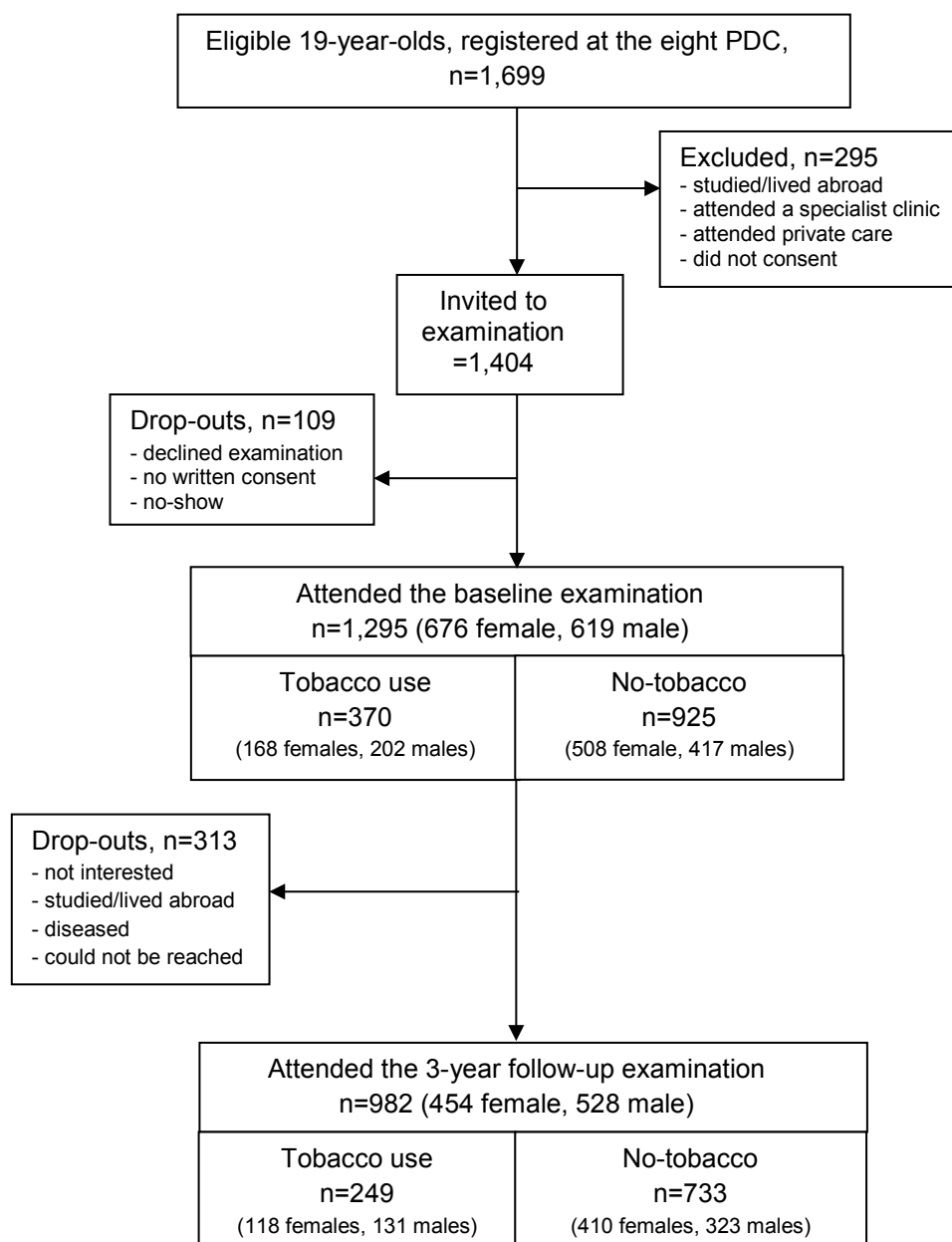

Supplement: Supplementary file 1 — Additional file 1. Flow-chart of the 3-year study with reasons for dropping out. [file 13104_2019_4253_MOESM1_ESM.pdf]
